# All-Cause Mortality and Cardiovascular Death between Statins and Omega-3 Supplementation: A Meta-Analysis and Network Meta-Analysis from 55 Randomized Controlled Trials

**DOI:** 10.3390/nu12103203

**Published:** 2020-10-20

**Authors:** Jeongseon Kim, Tung Hoang, Ji-Myung Kim, So Young Bu, Jeong-Hwa Choi, Eunju Park, Seung-Min Lee, Eunmi Park, Ji Yeon Min, In Seok Lee, So Young Youn, Jee-Young Yeon

**Affiliations:** 1 Department of Cancer Biomedical Science, National Cancer Center Graduate School of Cancer Science and Policy, Goyang 10408, Korea; 75256@ncc.re.kr; 2 Food and Nutrition Major, Division of Food Science and Culinary Arts, Shinhan University, Uijeongbu 11644, Korea; doublekim@hanmail.net; 3 Department of Food and Nutrition, Daegu University, Gyeongsan 38453, Korea; busy@daegu.ac.kr; 4 Department of Food and Nutrition, Keimyung University, Daegu 42601, Korea; jhchoi@kmu.ac.kr; 5 Department of Food and Nutrition, Kyungnam University, Changwon 51767, Korea; pej@kyungnam.ac.kr; 6 Department of Food and Nutrition, Yonsei University, Seoul 03722, Korea; leeseungmin@yonsei.ac.kr; 7 Department of Food and Nutrition, Hannam University, Daejeon 34430, Korea; eunmi_park@hnu.kr; 8 Dietetics and Nutrition Services Team, Asan Medical Center, Seoul 05505, Korea; sophiajym@hanmail.net; 9 Nutrition Support Team, Kyung Hee University Medical Center, Seoul 02447, Korea; inseok77@gmail.com; 10 Clinical Nutrition Part, Samsung Medical Center, Seoul 06351, Korea; soyoungy.youn@samsung.com; 11 Department of Food and Nutrition, Seowon University, Cheongju 28674, Korea; yeon_fn@naver.com

**Keywords:** cardiovascular disease, mortality, statin, omega-3, network meta-analysis

## Abstract

Statins and omega-3 supplementation have shown potential benefits in preventing cardiovascular disease (CVD), but their comparative effects on mortality outcomes, in addition to primary and secondary prevention and mixed population, have not been investigated. This study aimed to examine the effect of statins and omega-3 supplementation and indirectly compare the effects of statin use and omega-3 fatty acids on all-cause mortality and CVD death. We included randomized controlled trials (RCTs) from meta-analyses published until December 2019. Pooled relative risks (RRs) and 95% confidence intervals (CIs) were calculated to indirectly compare the effect of statin use versus omega-3 supplementation in a frequentist network meta-analysis. In total, 55 RCTs were included in the final analysis. Compared with placebo, statins were significantly associated with a decreased the risk of all-cause mortality (RR = 0.90, 95% CI = 0.86–0.94) and CVD death (RR = 0.86, 95% CI = 0.80–0.92), while omega-3 supplementation showed a borderline effect on all-cause mortality (RR = 0.97, 95% CI = 0.94–1.01) but were significantly associated with a reduced risk of CVD death (RR = 0.92, 95% CI = 0.87–0.98) in the meta-analysis. The network meta-analysis found that all-cause mortality was significantly different between statin use and omega-3 supplementation for overall population (RR = 0.91, 95% CI = 0.85–0.98), but borderline for primary prevention and mixed population and nonsignificant for secondary prevention. Furthermore, there were borderline differences between statin use and omega-3 supplementation in CVD death in the total population (RR = 0.92, 95% CI = 0.82–1.04) and primary prevention (RR = 0.85, 95% CI = 0.68–1.05), but nonsignificant differences in secondary prevention (RR = 0.97, 95% CI = 0.66–1.43) and mixed population (RR = 0.92, 95% CI = 0.75–1.14). To summarize, statin use might be associated with a lower risk of all-cause mortality than omega-3 supplementation. Future direct comparisons between statin use and omega-3 supplementation are required to confirm the findings.

## 1. Introduction

According to the Global Burden of Diseases 2017, an estimated 17.3 million (31%) deaths worldwide were caused by cardiovascular disease (CVD) [1]. It has been estimated that 125.6 million Americans will have CVD by 2030, an approximately 14% increase in prevalence from prior estimates in 2020 [2]. Although life expectancy increased by 7.4 years from 1990 to 2017 [3] and survival from CVD improved by 10.3% during 2007–2017 [1], the five-year mortality due to CVD remains as high as 13.7% in some age groups and populations [4].

To reduce CVD risk, lipid-lowering therapy such as statins has been recommended for the primary and secondary prevention of CVD [5,6,7]. Epidemiological evidence showed significantly reduced mortality-both from any cause and from CVD mortality-in statin-treated patients, with RRs (95% CI) of 0.89 (0.85–0.93) and 0.80 (0.71–0.91), respectively [8]. Among patients with established CVD, statins led to a statistically significant 12% reduction in CVD deaths compared to the control group (RR = 0.88, 95% CI = 0.81–0.96). However, statin-treated patients might also suffer from some harmful effects, including myopathy, diabetes, and hepatic dysfunction [8].

Furthermore, guidance on lifestyle management from the American College of Cardiology/American Heart Association (ACC/AHA) recommended a greater intake of fish or polyunsaturated fatty acids (particularly omega-3) as part of Mediterranean and Dietary Approaches to Stop Hypertension dietary patterns [9,10]. Omega-3 fatty acids can also be available as supplements that contain eicosapentaenoic acid (EPA) in combination with docosahexaenoic acid (DHA) or EPA only (with either ethyl or non-ethyl structures) [11]. A recent review from the AHA did not strongly recommend omega-3 supplementation to prevent CVD [12]. Higher intake of omega-3, regardless of the source, was found to have little or no effect on all-cause mortality (relative risk (RR) = 0.98, 95% confidence interval (CI) = 0.93–1.03) and CVD death (RR = 0.94, 95% CI = 0.89–1.00) [13]. However, updated findings from a recent meta-analysis showed that omega-3 supplementation was associated with a 7% lower risk of CVD death (RR = 0.93, 95% CI = 0.88–0.99) [14].

A recent study compared the effect of statins and omega-3 supplementation on risk reduction of CVD events including total CVD, coronary heart disease, myocardial infarction, and stroke [15]. However, such effects in terms of long-term outcomes such as mortality have not been elucidated. Additionally, the pooled estimates were not specified for primary and secondary prevention population and the dose–response relationship was not investigated. Therefore, we conducted this study to examine the dose–response effect of statins and omega-3 supplementation in different study population. Furthermore, given that the most recent updated evidence reported significant effects of omega-3 supplementation [14], which has fewer side effects than statins, we conducted this meta-analysis of randomized controlled trials (RCTs) to estimate the relative effects of statin use and omega-3 supplementation compared with placebo and performed a network meta-analysis (NMA) to estimate the comparative effects of statin use versus omega-3 supplementation on all-cause mortality and CVD death. 

## 2. Materials and Methods 

### 2.1. Search Strategy

We used PubMed to retrieve systematic reviews with or without meta-analysis. On 22 December 2019, the following terms were searched without language restriction: statin, omega-3 supplementation, cardiovascular disease, systematic review, and meta-analysis. The RCTs from relevant systematic reviews were then assessed for eligibility criteria. We incidentally searched for relevant RCTs published after the cut-off date for inclusion in previous systematic reviews. 

### 2.2. Study Eligibility

The inclusion criteria for studies to be evaluated in the final analysis were as follows: (i) statins or omega-3 supplementation were compared with or added to a placebo; (ii) the sample size and the number of all-cause mortality and CVD death events were reported; and (iii) the follow-up duration was at least one year. RCTs were excluded if the source of omega-3 was from dietary intake or the comparison arm contained omega-6 fatty acids.

Two investigators (T.H. and J.K.) independently assessed articles for the inclusion and exclusion criteria and were responsible for data extraction. Any discrepancies were discussed and resolved through consultations with other investigators (J.-M.K., S.Y.B, and J.-H.C.). Details were recorded about the authors’ name; publication year; country; recruitment period; the mean or median follow-up time; body mass index (BMI); demographic information on age and sex; history of CVD, coronary heart disease, myocardial infarction, heart failure, hypertension, dyslipidemia, and diabetes; the mean or median number of smokers and obese subjects; sample size; the daily dose of the intervention; and the number and percentage of outcome events for each treatment arm.

### 2.3. Statistical Analyses

We conducted both direct and indirect comparisons for all-cause mortality and CVD death. In the direct comparison, we investigated the effects of statins or omega-3 supplementation compared with the placebo in a random-effects model using the DerSimonian–Laird method [16]. We additionally performed subgroup analyses by type of prevention, type of statin, and type of omega-3 supplementation. In particular, RCTs in which at least 80% of the study population had any CVD risk factors (hypertension, dyslipidemia, diabetes, smoking, or obesity) were considered to be conducted for primary prevention, whereas those in which at least 80% of the study population had any history of CVD events (CVD, coronary heart disease, myocardial infarction, and heart failure) were considered to be conducted for secondary prevention. The remaining RCTs were considered as mixed population. In the indirect comparison, pooled RRs and 95% CIs were calculated to examine the pairwise comparisons of statins versus placebo, omega-3 supplementation versus placebo, and statins versus omega-3 supplementation in an NMA using a frequentist approach [17].

Heterogeneity across studies was measured by calculating the Higgins I^2^ [18]. Substantial heterogeneity was considered to be present if the I^2^ value was greater than 50% or the *p*-value was less than 0.05. Evidence of asymmetry and publication bias was assessed using Begg funnel plots [19] and the Egger test [20], in line with recent recommendations [21]. Publication bias was considered to be present if the funnel plot was asymmetric or if the *p*-value from the Egger test was lower than 0.05. In this case, pooled estimates from the fixed-effects model would be reported along with those from the random-effects model to counterweight the possible inflation of the therapeutic effect among large and small individual RCTs. For dose–response meta-analysis, the correlated RR estimates across different doses of statins and omega-3 supplementation were calculated using the generalized least-square regression method.

All statistical analyses were performed using Stata SE version 14.0 (StataCorp, College Station, TX, USA).

## 3. Results

### 3.1. Literature Search

**Figure 1:**
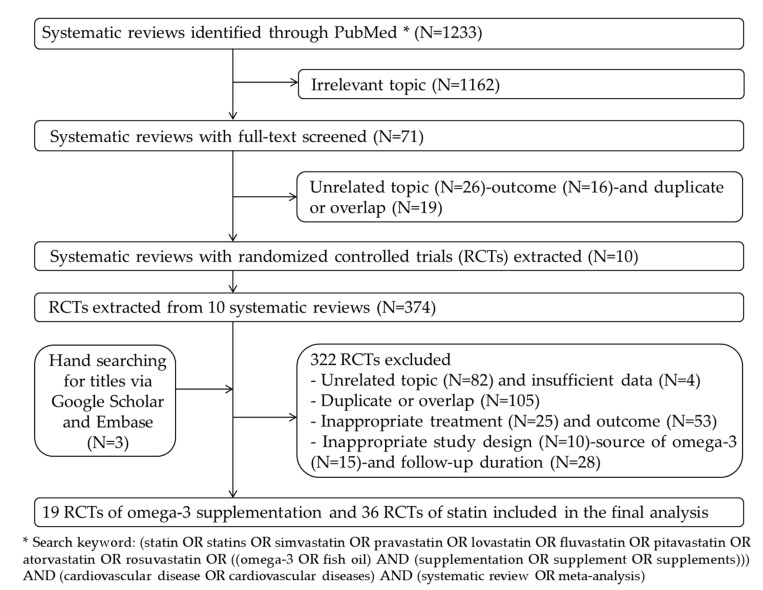
Flowchart of search strategy and study selection.

We identified 1233 articles in the database search (Figure 1). After irrelevant records were removed, 71 full texts were screened. Of these, 61 were discarded because they addressed irrelevant topics (*n* = 26), reported inappropriate outcomes (*n* = 16), or were duplicate or overlapping (*n* = 19). From the remaining 10 systematic reviews and meta-analyses, 374 RCTs were extracted and accessed to evaluate them for the eligibility criteria. After additionally hand-searching for updated RCTs (*n* = 3) and excluding ineligible RCTs (*n* = 322), 55 studies [5,22,23,24,25,26,27,28,29,30,31,32,33,34,35,36,37,38,39,40,41,42,43,44,45,46,47,48,49,50,51,52,53,54,55,56,57,58,59,60,61,62,63,64,65,66,67,68,69,70,71,72,73,74,75,76] with 36 RCTs of statins and 19 RCTs of omega-3 supplementation were included in the final meta-analysis and NMA.

### 3.2. Descriptive Characteristics

Table A1, Table A2 and Table A3 summarize the characteristics of the studies included in the final analysis. In total, 278,954 participants were assigned to receive a statin (*n* = 73,676), omega-3 supplementation (*n* = 65,819), or a placebo (*n* = 139,459). The median age, percentage of male subjects, follow-up duration, and BMI across studies was 52.5 years old, 74.4%, 3.9 years, and 27 kg/m^2^, respectively. The following distribution was found for patients with a history of various conditions: CVD, 18.5%; coronary heart disease, 13.0%; myocardial infarction, 24.2%; heart failure, 3.5%; hypertension, 46.0%; dyslipidemia, 37.5%; diabetes, 19.2%; smoking, 40.4%; and obesity, 37.1%.

In the primary prevention population, 68,101 participants were assigned to receive a statin (*n* = 8908), omega-3 supplementation (*n* = 25,149), or a placebo (*n* = 34,044). The median age, percentage of male subjects, follow-up duration, and BMI across studies were 63.15 years old, 67.15%, four years, and 28.75 kg/m^2^, respectively. The following distribution was found for patients with a history of CVD risk factors: hypertension, 55.1%; dyslipidemia, 71.2%; diabetes, 79.95%; smoking, 47.1%; and obesity, 47.55%.

In the secondary prevention population, 19,805 participants were assigned to receive a statin (*n* = 3996), omega-3 supplementation (*n* = 5898), or a placebo (*n* = 9911). The median age, percentage of male subjects, follow-up duration, and BMI across studies were 68 years old, 78.2%, 3.4 years, and 27 kg/m^2^, respectively. The following distribution was found for patients with a history of CVD events: coronary heart disease, 100%; myocardial infarction, 60%; and heart failure, 100%.

In the mixed population, 191,048 participants were assigned to receive a statin (*n* = 60,772), omega-3 supplementation (*n* = 34,772), or a placebo (*n* = 95,504). The median age, percentage of male subjects, follow-up duration, and BMI across studies were 62.05 years old, 71.2%, 3.65 years, and 26.6 kg/m^2^, respectively. The following distribution was found for patients with a history of various conditions: CVD, 18.5%; coronary heart disease, 12.95%; myocardial infarction, 18.85%; heart failure, 0%; hypertension, 41.8%; dyslipidemia, 36.8%; diabetes, 14.8%; smoking, 42.2%; and obesity, 20.55%.

### 3.3. Meta-Analysis 

**Figure A1:**
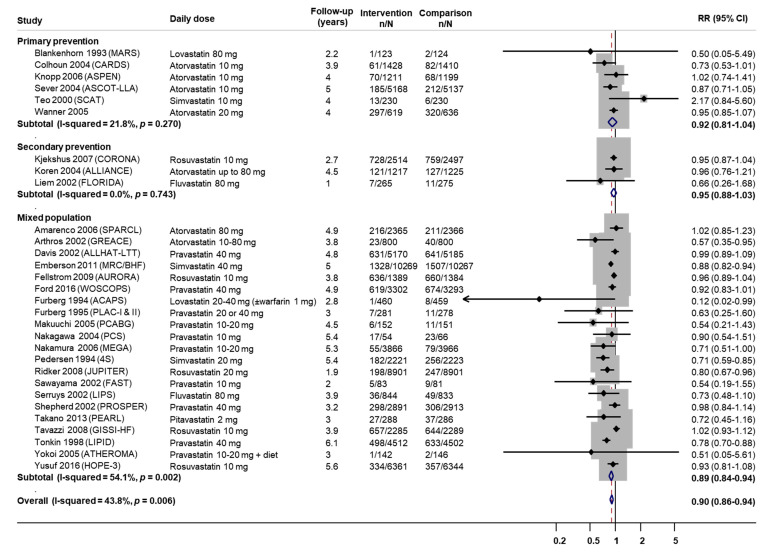
Subgroup meta-analysis for the effect of statin use on primary or secondary prevention of all-cause mortality. Full black quadrilateral represents the point estimate of individual studies and blank with quadrilateral represents pooled estimates. RR, relative risk; CI, confidence interval. Pooled estimates for the overall study population and subgroups are highlighted with bold font.

**Figure A2:**
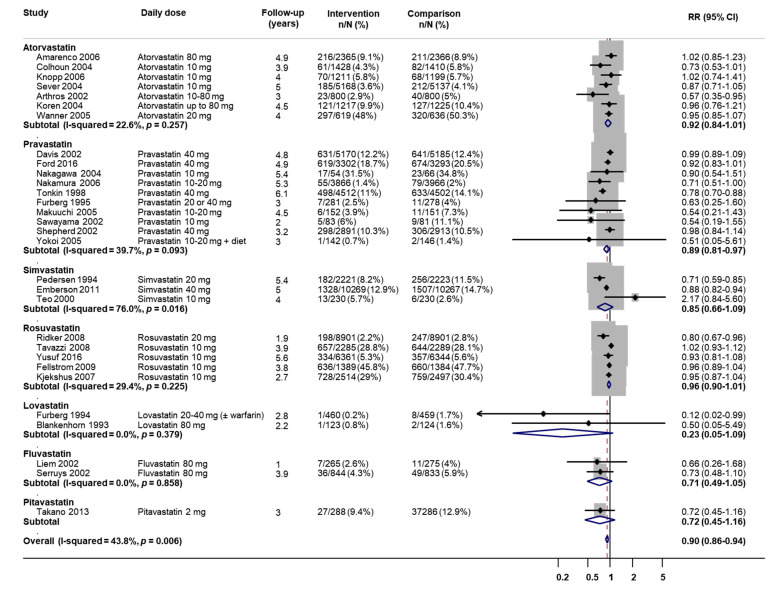
Subgroup meta-analysis for the effect of different statins on all-cause mortality. Full black quadrilateral represents the point estimate of individual studies and blank with quadrilateral represents pooled estimates. RR, relative risk; CI, confidence interval. Pooled estimates for the overall study population and subgroups are highlighted with bold font.

**Figure A3:**
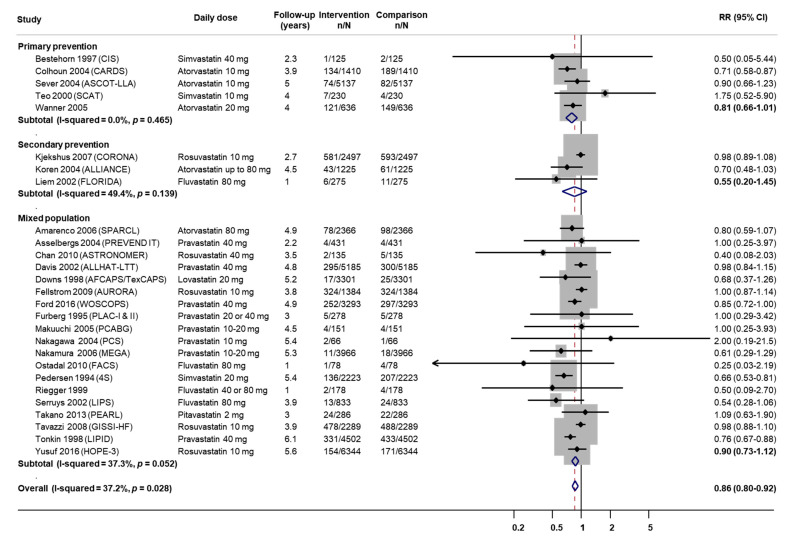
Subgroup meta-analysis for the effect of statin use on primary or secondary prevention of cardiovascular disease death. Full black quadrilateral represents the point estimate of individual studies and blank with quadrilateral represents pooled estimates. RR, relative risk; CI, confidence interval. Pooled estimates for the overall study population and subgroups are highlighted with bold font.

**Figure A4:**
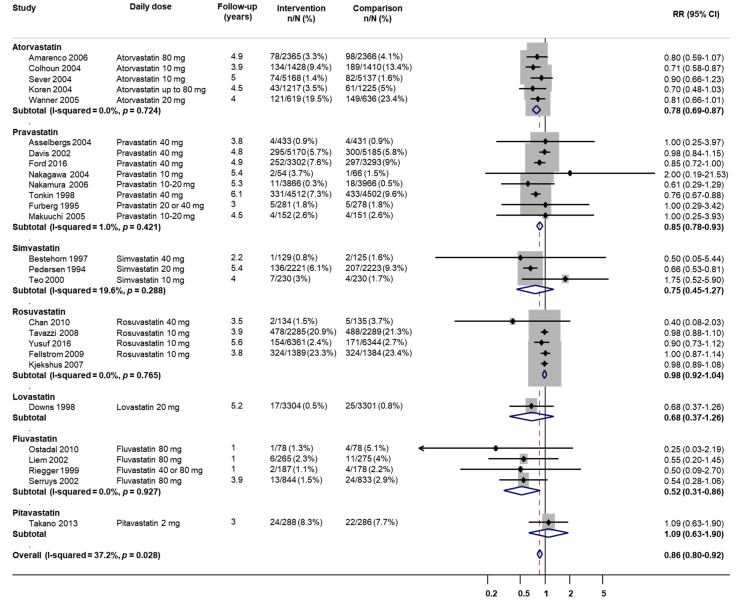
Subgroup meta-analysis for the effect of different statins on cardiovascular disease death. Full black quadrilateral represents the point estimate of individual studies and blank with quadrilateral represents pooled estimates. RR, relative risk; CI, confidence interval. Pooled estimates for the overall study population and subgroups are highlighted with bold font.

**Figure A5:**
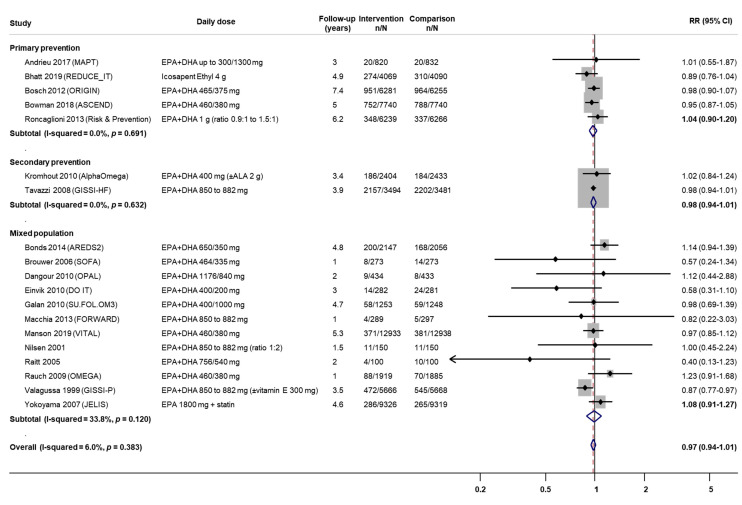
Subgroup meta-analysis for the effect of omega-3 supplementation on primary or secondary prevention of all-cause mortality. Full black quadrilateral represents the point estimate of individual studies and blank with quadrilateral represents pooled estimates. RR, relative risk; CI, confidence interval; EPA, eicosapentaenoic acid; DHA, docosahexaenoic acid. ALA, alpha-linolenic acid. Pooled estimates for the overall study population and subgroups are highlighted with bold font.

**Figure A6:**
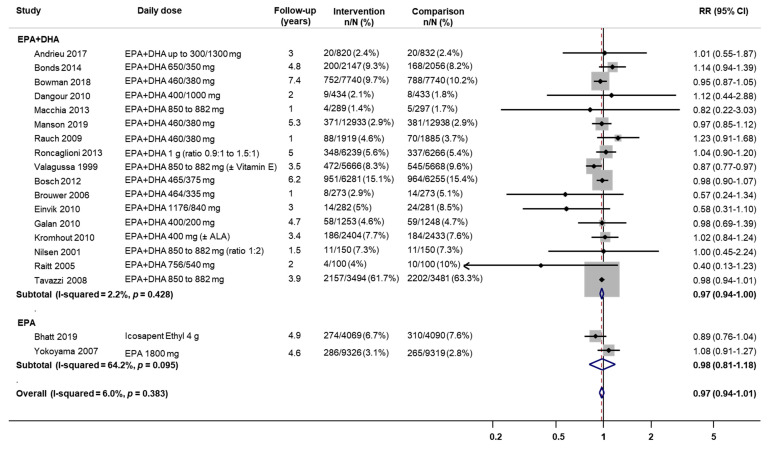
Subgroup meta-analysis for the effect of different omega-3 supplementation types on all-cause mortality. Full black quadrilateral represents the point estimate of individual studies and blank with quadrilateral represents pooled estimates. RR, relative risk; CI, confidence interval; EPA, eicosapentaenoic acid; DHA, docosahexaenoic acid. ALA, alpha-linolenic acid. Pooled estimates for the overall study population and subgroups are highlighted with bold font.

**Figure A7:**
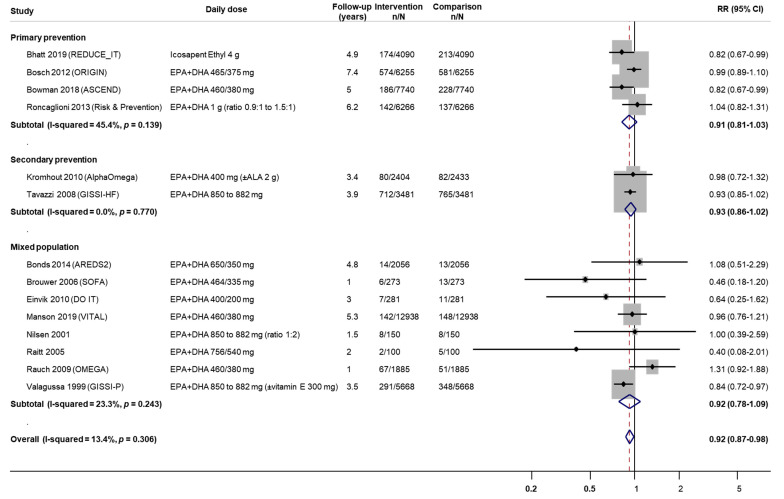
Subgroup meta-analysis for the effect of omega-3 supplementation on primary or secondary prevention of cardiovascular disease death. Full black quadrilateral represents the point estimate of individual studies and blank with quadrilateral represents pooled estimates. RR, relative risk; CI, confidence interval; EPA, eicosapentaenoic acid; DHA, docosahexaenoic acid. ALA, alpha-linolenic acid. Pooled estimates for the overall study population and subgroups are highlighted with bold font.

**Figure A8:**
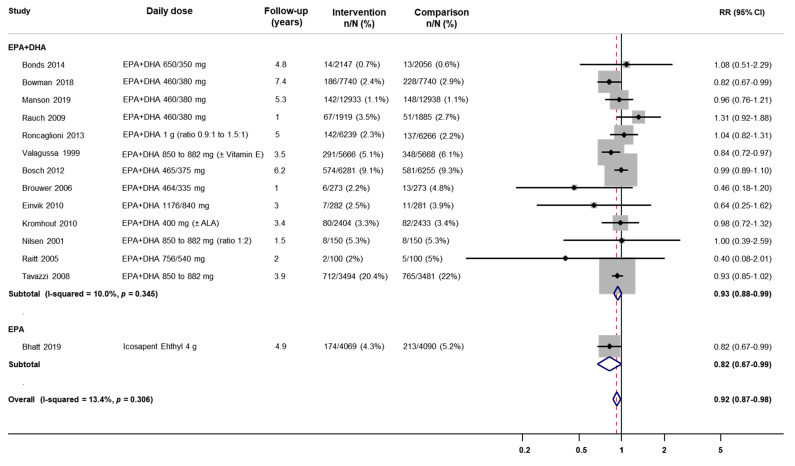
Subgroup meta-analysis for the effect of different omega-3 supplementation types on cardiovascular disease death. Full black quadrilateral represents the point estimate of individual studies and blank with quadrilateral represents pooled estimates. RR, relative risk; CI, confidence interval; EPA, eicosapentaenoic acid; DHA, docosahexaenoic acid. ALA, alpha-linolenic acid. Pooled estimates for the overall study population and subgroups are highlighted with bold font.

**Figure A9:**
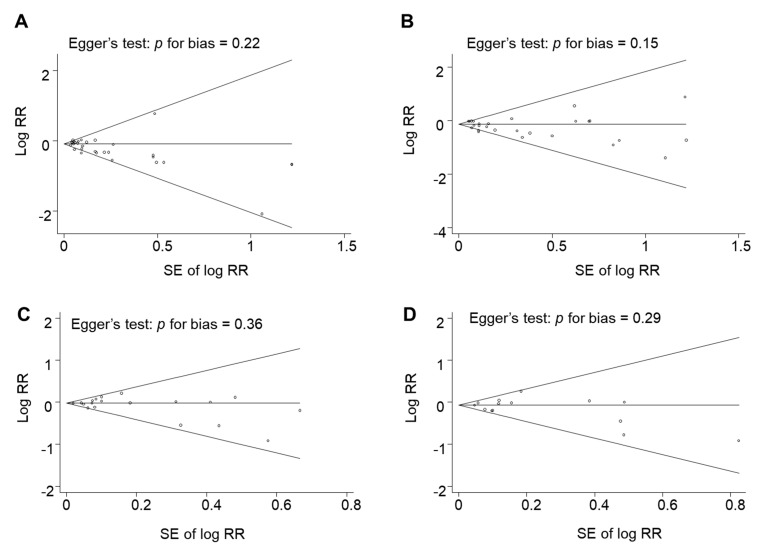
Funnel plot analysis to detect publication bias: (**A**) statins and all-cause mortality; (**B**) statins and cardiovascular disease death; (**C**) omega-3 supplementation and all-cause mortality; and (**D**) omega-3 supplementation and cardiovascular disease death). RR, relative risk; SE, standard error.

Direct comparisons of statin use or omega-3 supplementation versus placebo in terms of mortality outcomes are presented in Table 1 and Figure A1, Figure A2, Figure A3, Figure A4, Figure A5, Figure A6, Figure A7 and Figure A8. Overall, statin use showed a statistically significant risk reduction for all-cause mortality and CVD death, with RRs (95% CIs) 0.90 (0.86–0.94) and 0.86 (0.80–0.92), respectively, whereas the findings for omega-3 supplementation were of borderline significance, with RRs (95% CIs) of 0.97 (0.94–1.01) and 0.92 (0.87–0.98), respectively. Figure A9 shows that no publication bias was detected for the association between statin use and all-cause mortality (*p* = 0.22) or CVD death (*p* = 0.15). Similarly, no publication bias was detected for the association between omega-3 supplementation and all-cause mortality (*p* = 0.36) or CVD death (*p* = 0.29). 

In the subgroup analysis by type of prevention, statins was observed to reduce all-cause mortality for the mixed population (RR = 0.89, 95% CI = 0.84–0.94), but borderline for primary (RR = 0.92, 95% CI = 0.81–1.04) and secondary prevention (RR = 0.95, 95% CI = 0.88–1.03). However, the CVD death reduction effect of statins was not observed for secondary prevention (RR = 0.81, 95% CI = 0.66–1.01) and mixed population (RR = 0.90, 95% CI = 0.73–1.12), but borderline for primary prevention (RR = 0.81, 95% CI = 0.66–1.01).

In the subgroup analysis by type of statins, risk reduction effects were only observed for pravastatin in terms of all-cause mortality and atorvastatin, pravastatin, fluvastatin, and pitavastatin in terms of CVD death. In contrast, the subgroup analysis by type of omega-3 fatty acids showed similar findings to those of the overall intervention. The pooled estimates and level of heterogeneity did not change much when excluding the REDUCE-IT trial, in which icosapentyl ethyl was used at a high dose of 4 g.

**Figure A10:**
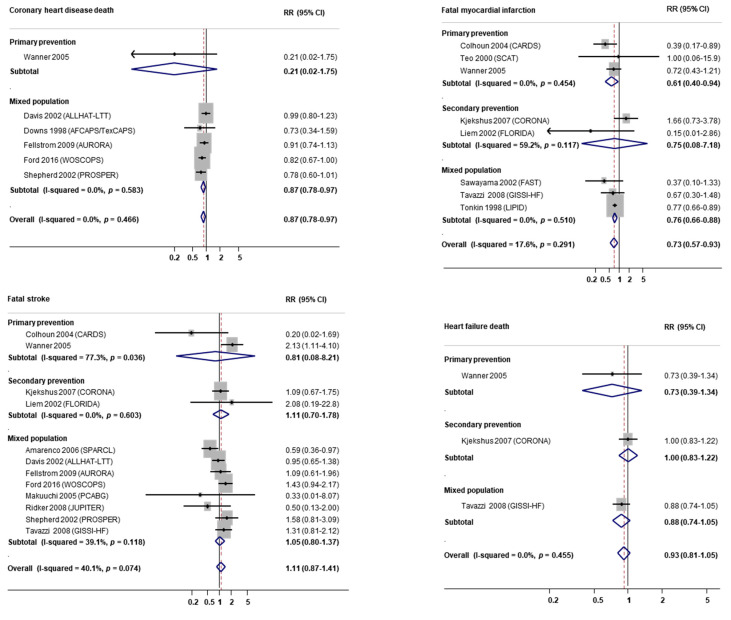
Subgroup meta-analysis for the effect of statins on specific causes of cardiovascular disease death. Full black quadrilateral represents the point estimate of individual studies and blank with quadrilateral represents pooled estimates. RR, relative risk; CI, confidence interval. Pooled estimates for the overall study population and subgroups are highlighted with bold font.

**Figure A11:**
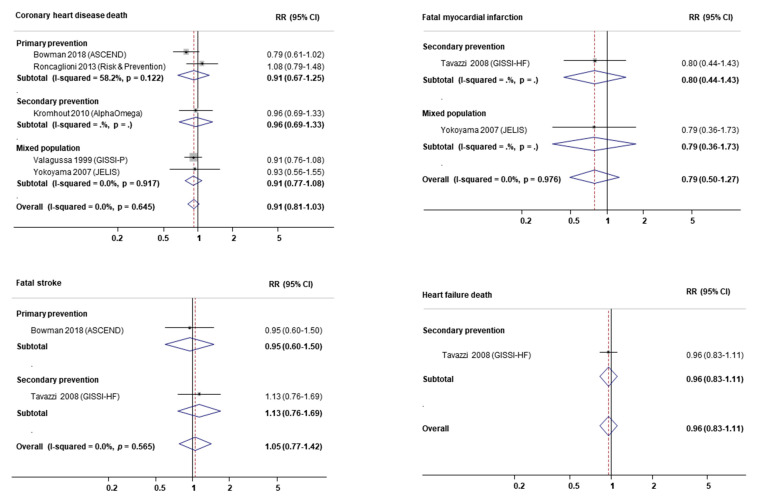
Subgroup meta-analysis for the effect of omega-3 supplementation on specific causes of cardiovascular disease death. Full black quadrilateral represents the point estimate of individual studies and blank with quadrilateral represents pooled estimates. RR, relative risk; CI, confidence interval. Pooled estimates for the overall study population and subgroups are highlighted with bold font.

Additional analyses by specific causes of death due to CVD found that statins were associated with lower risks of coronary heart disease death (RR = 0.87, 95% CI = 0.78–0.97) and fatal myocardial infarction (RR = 0.73, 95% CI = 0.57–0.93), but nonsignificant results for fatal stroke and heart failure death (Figure A10). In contrast, omega-3 supplementation was not associated with any specific causes of CVD death (Figure A11).

**Figure A12:**
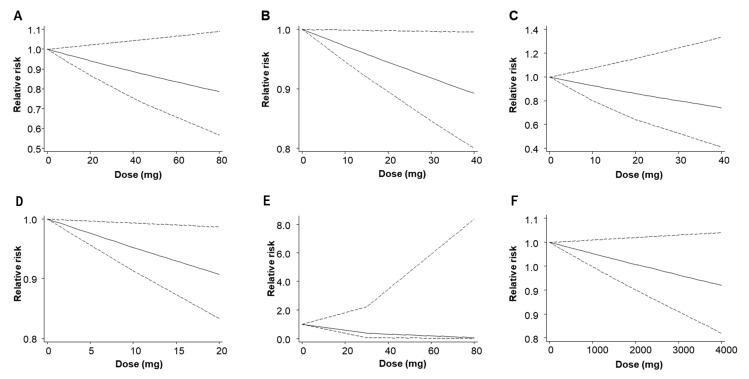
Dose–response meta-analysis of the association between (**A**) atorvastatin, (**B**) pravastatin, (**C**) simvastatin, (**D**) rosuvastain, (**E**) lovastatin, and (**F**) omega-3 supplementation with all-cause mortality.

**Figure A13:**
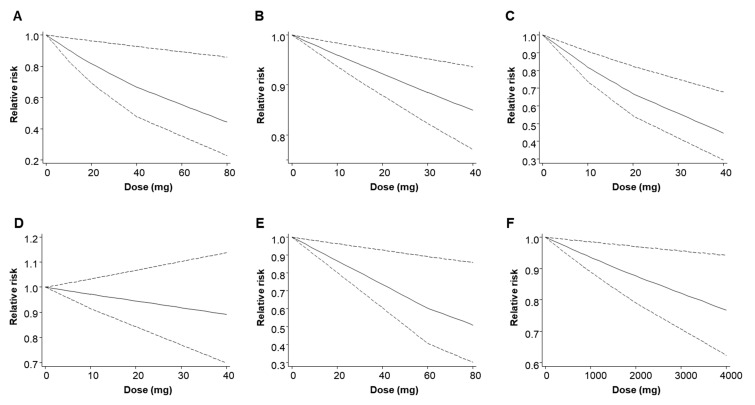
Dose–response meta-analysis of the association between (**A**) atorvastatin, (**B**) pravastatin, (**C**) simvastatin, (**D**) rosuvastain, (**E**) fluvastatin, and (**F**) omega-3 supplementation with cardiovascular death.

Dose–response meta-analysis for the effect of statins and omega-3 supplementation on all-cause mortality and CV death is summarized in Table A4 and Figure A12 and Figure A13. There was a dose–response relationship between rosuvastatin and all-cause mortality, with a 9% decrement in all-cause mortality (per 10-mg RR = 0.91, 95% CI = 0.80–0.99). There was also dose–response relationship between atorvastatin, pravastatin, simvastatin, fluvastatin, and omega-3 supplementation and CVD death, with per 10-mg RRs (95% CI) of 0.90 (0.83–0.98), 0.96 (0.94–0.98), 0.82 (0.74–0.91), and 0.92 (0.86–0.98) for statins and per 1000-mg RR (95% CI) of 0.94 (0.89–0.99) for omega-3 supplementation.

### 3.4. Network Meta-Analysis

An NMA combining direct and indirect estimates for pairwise comparisons among omega-3 supplementation, statins, and placebo is shown in Table 2. Similar to findings from the meta-analysis, omega-3 supplementation showed a lower risk of all-cause mortality than placebo, but not to a significant extent (RR = 0.97, 95% CI = 0.92–1.03), whereas randomization to omega-3 supplementation reduced CVD death by 9% compared to placebo (RR = 0.91, 95% CI = 0.84–0.99). Statins also demonstrated significant risk reductions for all-cause mortality and CVD death of 12% and 16%, with RRs (95% CIs) of 0.88 (0.84–0.93) and 0.84 (0.78–0.81), respectively. A significant risk reduction was observed for the effects of statin use versus omega-3 supplementation on all-cause mortality (RR = 0.91, 95% CI = 0.85–0.98) but borderline on CVD death (RR = 0.92, 95% CI = 0.82–1.04).

In the subgroup analysis of primary prevention, there were borderline effects of statin use and omega-3 supplementation on all-cause mortality (RR = 0.92, 95% CI = 0.80–1.06) and CVD death (RR = 0.85, 95% CI = 0.68–1.05). However, nonsignificant differences were observed for both all-cause mortality and CVD death in the subgroup analysis of secondary prevention, with RRs (95% CIs) of 0.98 (0.85–1.13) and 0.97 (0.66–1.43), respectively. In the subgroup analysis of mixed population, statin use was found to show borderline effect on all-cause mortality in the comparison with omega-3 supplementation (RR = 0.88, 95% CI = 0.77–1.01), whereas the effect on CVD death was not significantly different (RR = 0.92, 95% CI = 0.75–1.14).

## 4. Discussion

We performed a systematic review, meta-analysis, and NMA to summarize the current evidence on the effects of statin use and omega-3 supplementation on mortality outcomes. In this study, analyses of statins showed a statistically significant 10% reduction in the risk of all-cause mortality and a 14% reduction in the risk of CVD death. Additionally, the meta-analysis showed that omega-3 supplementation did not lead to a significantly lower risk of all-cause mortality, whereas the risk of CVD death was reduced significantly by 8%. In the comparison with omega-3 supplementation, statins were found to significantly reduce all-cause mortality in the total population, borderline in the subgroup of primary prevention and mixed population, but not in the subgroup of secondary prevention. Furthermore, the NMA showed that the effects of statins and omega-3 supplementation on CVD death were borderline in the total population and in the subgroup of primary prevention, but not in the subgroups of secondary prevention and mixed population.

Our findings for the effect of statins are consistent with the latest updated meta-analysis, in which 11% and 20% risk reductions for all-cause mortality (24 RCTs) and CVD death (15 RCTs) were observed with the intervention of statins [8]. More RCTs were included in the current meta-analysis, with a similar effect on all-cause mortality (29 RCTs, 10% versus 11% risk reduction) but a smaller effect on CVD death (26 RCTs, 14% versus 20% risk reduction), although no statistical test was performed to compare the difference. Statins can change plasma levels of total cholesterol (TC), triglyceride (TG), high-density lipoprotein (HDL-C), and low-density lipoprotein (LDL-C) by inhibiting the synthesis of cholesterol in the liver by 3-hydroxy-3-methylglutaryl-CoA (HMG-CoA) reductase [77,78,79,80,81,82,83], thereby reducing the risk of CVD. However, most of the individual RCTs did not detect a significantly beneficial effect of statins on mortality outcomes. Randomization to statins might have led to a decrease in both all-cause mortality and CVD death in the LIPID and 4S trials [48,84]. More additional RCTs observed a significant effect of statins on all-cause mortality than on CVD death [26,37,41,60], but the effect was closer to the null. Since more RCTs found a significant effect, substantial heterogeneity remained among RCTs investigating the association between statin use and all-cause mortality (I^2^ = 43.8%, *p* = 0.006). Furthermore, recent studies found that statin use was associated with a lower risk of mortality in patients receiving clopidogrel (RR = 0.54, 95% CI = 0.40–0.74) [85] and Asian patients with type 2 diabetes [86]. Findings from another prospective cohort study of nearly 86,000 participants also supported the beneficial effect of statins on all-cause mortality (hazard ratio 0.86, 95% CI = 0.77–0.95) and CVD death (hazard ratio 0.75, 95% CI = 0.64–0.89) [87]. Moreover, our additional analyses by specific causes of death by CVD suggested the potential effect of statins on risk reductions of coronary heart disease death and fatal myocardial infarction.

The findings are in line with a recent meta-analysis by Abdelhamid et al., who reported that higher intake of omega-3 fatty acids had little or no effect on all-cause mortality and CVD death [13]. Apart from a different methodological approach, we included more up-to-date RCTs in this study, but excluded several RCTs with issues that might cause discrepancies. Several lipid biomarkers, including TC, TG, HDL-C, and LDL-C have been reported to be associated with CVD risk [88,89,90,91]. Omega-3 fatty acids can decrease plasma TG levels by reducing the production of hepatic very-low-density lipoprotein cholesterol and increasing chylomicron clearance. Furthermore, omega-3 fatty acids can change dysfunctional HDL-C to functional HDL-C and also exert weak TC- and LDL-C lowering effects [92,93,94]. In contrast with the findings of the pooled analysis, most of the individual RCTs did not find that omega-3 supplementation led to a significant reduction of mortality outcomes. The results of the GISSI-P trial stand apart from the null findings of 19 RCTs that examined the association between omega-3 supplementation and all-cause mortality, with an RR of 0.87 (95% CI = 0.77–0.97) (Figure A1 and Figure A3) [40]. Therefore, the pooled effect size for the effect of omega-3 supplementation on all-cause mortality was non-significantly different, as expected. In terms of CVD death, the results from the REDUCE-IT [40], ASCEND [24], and GISSI-P [40] trials, which accounted for 31.4% of the sample size, were only borderline significant, with RRs and 95% CIs of 0.82 (0.67–0.99), 0.82 (0.67–0.99), and 0.84 (0.72–0.97), respectively (Figure A2 and Figure A4). However, the pooled analysis with a large sample showed that omega-3 supplementation had a significant effect on CVD death, with low heterogeneity (I^2^ = 6.0%, *p* = 0.38). Furthermore, subgroup analyses by specific causes of death due to CVD showed no significant associations between omega-3 supplementation and the deaths of coronary heart disease, myocardial infarction, stroke, and heart failure. 

In this study, we found a significantly lower risk of all-cause mortality in the statin group than the omega-3 supplementation group in primary prevention but not in secondary prevention. Because we defined studies of primary prevention as those with high probabilities of CVD risk factors, statin use was therefore hypothesized to reduce the death of CVD risk factor-related diseases. Moreover, statins were observed for their beneficial effects on reducing the mortality from breast, colorectal, kidney, ovarian, and prostate cancers, which contributed to all-cause mortality [95]. However, those effects of omega-3 supplementation have not been adequately investigated. In contrast, the effect of statins and omega-3 supplementation on CVD death reduction was borderline only. The mechanism is unclear, but a possible explanation may be found in the overlap of the pleiotropic effects of statins with the actions of omega-3 supplementation with, including endothelial function improvement, anti-thrombotic effects, and antioxidant effects [96]. Although previous RCTs and meta-analysis examined the effect of combination therapy with statins and omega-3 supplementation versus statins alone in patients with dyslipidemia [97,98,99] or cardiovascular events [92], the effects of statins and omega-3 supplementation have not been investigated. Our methodology was designed to minimize variety in the placebo group, which is the mediating factor in indirect comparisons between statins and omega-3 supplementation. We excluded RCTs if the placebo group contained omega-6 or even low doses of omega-3 to avoid bias in our pooled estimates. Furthermore, a subgroup analysis by primary and secondary prevention was performed to obtain robust findings. 

Despite its strengths, there are certain limitations of our study. First, considering the nature of the study, potential heterogeneity in the associations with CVD death remained. Second, due to the lack of data, we were unable to perform the sex-specific meta-analysis to test whether there were different effects in males and females. Lastly, although both EPA alone [75] and the highly purified and stable EPA ethyl ester [28] have been determined to have beneficial effects, a pooled analysis with the other form of EPA + DHA might have introduced heterogeneity due to diversity. Combinations of different statin types might have also led to heterogeneity. Nevertheless, this is an up-to-date study with 278,954 participants that compared the effects of statin use and omega-3 supplementation on mortality outcomes. 

## 5. Conclusions

In summary, statin use was significantly associated with decreased risks of mortality outcomes, whereas omega-3 supplementation showed nonsignificant or little effect on all-cause mortality and CVD death. Statin use was shown to be more effective in reducing all-cause mortality than omega-3 supplementation. However, the effect was borderline in terms of CVD mortality. Future direct comparisons between omega-3 supplementation and statin use are required to detect the statistical benefits of omega-3 supplementation.

## Figures and Tables

**Table 1:** Meta-analysis of the associations between statins and omega-3 supplementation with all-cause mortality and cardiovascular death.

Intervention	All-Cause Mortality		Cardiovascular Disease Death	
	No. Studies (I^2^)	RR (95% CI)	No. Studies (I^2^)	RR (95% CI)
**Statins**				
Overall	30 (43.8%)	**0.90 (0.86–0.94)**	27 (37.2%)	**0.86 (0.80–0.92)**
Primary prevention	5 (21.8%)	0.92 (0.81–1.04)	5 (0%)	0.81 (0.66–1.01)
Secondary prevention	3 (0%)	0.95 (0.88–1.03)	3 (49.4%)	0.55 (0.20–1.45)
Mixed population	21 (54.1%)	**0.89 (0.84–0.94)**	19 (37.2%)	0.90 (0.73–1.12)
Atorvastatin	7 (22.6%)	0.92 (0.84–1.01)	5 (0%)	**0.78 (0.69–0.87)**
Pravastatin	10 (39.7%)	**0.89 (0.81–0.97)**	8 (1.0%)	**0.85 (0.78–0.93)**
Simvastatin	3 (76.0%)	0.85 (0.66–1.09)	3 (19.6%)	0.75 (0.45–1.27)
Rosuvastatin	5 (29.4%)	0.96 (0.90–1.01)	5 (0%)	0.98 (0.92–1.04)
Lovastatin	2 (0%)	0.23 (0.05–1.09)	1 (NA)	0.68 (0.92–1.26)
Fluvastatin	2 (0%)	0.71 (0.49–1.05)	4 (0%)	**0.52 (0.31–0.86)**
Pitavastatin	1 (NA)	0.72 (0.45–1.16)	1 (NA)	**0.52 (0.31–0.86)**
**Omega-3 supplementation (including REDUCE-IT trial)**				
Overall	19 (6.0%)	0.97 (0.94–1.01)	14 (13.4%)	**0.92 (0.87–0.98)**
Primary prevention	5 (0%)	1.04 (0.90–1.20)	4 (45.4%)	0.91 (0.81–1.03)
Secondary prevention	2 (0%)	0.98 (0.94–1.01)	2 (0%)	0.93 (0.86–1.02)
Mixed population	12 (33.8%)	1.09 (0.91–1.27)	8 (23.3%)	0.92 (0.78–1.09)
EPA+DHA	17 (2.2%)	0.97 (0.94–1.00)	13 (10.0%)	**0.93 (0.88–0.99)**
EPA only	2 (6.0%)	0.98 (0.81–1.18)	1 (NA)	**0.82 (0.67–0.99)**
**Omega-3 supplementation (excluding REDUCE-IT trial)**				
Overall	18 (4.4%)	0.98 (0.95–1.01)	13 (10.0%)	**0.93 (0.88–0.99)**
Primary prevention	4 (0%)	0.98 (0.93–1.04)	3 (42.2%)	0.95 (0.83–1.08)
Secondary prevention	2 (0%)	0.98 (0.94–1.01)	2 (0%)	0.93 (0.86–1.02)
Mixed population	12 (33.8%)	1.09 (0.91–1.27)	8 (23.3%)	0.92 (0.78–1.09)
EPA + DHA	17 (2.2%)	0.97 (0.94–1.00)	13 (10.0%)	**0.93 (0.88–0.99)**
EPA only	1 (NA)	1.08 (0.91–1.27)	-	-

RR, relative risk; CI, confidence interval; EPA, eicosapentaenoic acid; DHA, docosahexaenoic acid; NA, not applicable; REDUCE-IT, Reduction of Cardiovascular Events with Icosapent Ethyl - Intervention Trial. Bold font indicates statistical significance.

**Table 2:** Relative risks and 95% confidence intervals from network meta-analysis of omega-3 supplementation, statins, and placebo effects on all-cause mortality and cardiovascular disease death.

Comparison	All-Cause Mortality	Cardiovascular Disease Death
**Overall**		
Statins vs. placebo	**0.88 (0.84–0.93)**	**0.84 (0.78–0.91)**
Omega-3 vs. placebo	0.97 (0.92–1.03)	**0.91 (0.84–0.99)**
Statins vs. omega-3	**0.91 (0.85–0.98)**	0.92 (0.82–1.04)
**Primary prevention**		
Statins vs. placebo	0.89 (0.78–1.01)	**0.77 (0.65–0.93)**
Omega-3 vs. placebo	0.97 (0.91–1.03)	0.91 (0.81–1.03)
Statins vs. omega-3	0.92 (0.80–1.06)	0.85 (0.68–1.05)
**Secondary prevention**		
Statins vs. placebo	0.93 (0.84–1.04)	0.90 (0.66–1.22)
Omega-3 vs. placebo	0.95 (0.87–1.04)	0.93 (0.78–1.10)
Statins vs. omega-3	0.98 (0.85–1.13)	0.97 (0.66–1.43)
**Mixed population**		
Statins vs. placebo	**0.87 (0.81–0.93)**	**0.84 (0.76–0.93)**
Omega-3 vs. placebo	0.98 (0.88–1.10)	0.91 (0.76–1.09)
Statins vs. omega-3	0.88 (0.77–1.01)	0.92 (0.75–1.14)

Bold font indicates statistical significance.

## Appendix A

**Table A1:** General characteristics of the included randomized controlled trials.

Study	Countries	Period	Sample Size	Age (Years)	Male	Follow-Up (Years)	Body Mass Index (kg/m^2^)
**Statins**							
**Primary prevention**							
Bestehorn 1997 (CIS)	Germany	1989–1992	254	49.8	254 (100%)	2.3	
Blankenhorn 1993 (MARS)		1985–1989	247	58	225 (91%)	2.2	
Colhoun 2004 (CARDS)	United Kingdom and Ireland	1997–2001	2838	62	1930 (68%)	3.9	
Knopp 2006 (ASPEN)	14 countries	1996–1999	2410	61	1598 (66.3%)	4	28.9
Sever 2004 (ASCOT-LLA)	United Kingdom and Ireland	1998–2000	10305	63	8347 (81%)	5	28.6
Teo 2000 (SCAT)	Canada	1996–1998	460	61	409 (89%)	4	
Wanner 2005	Germany	1998–2002	1255	65.7	676 (53.9%)	4	27.5
**Secondary prevention**							
Kjekshus 2007 (CORONA)	21 countries	2003–2005	5011	73	3808 (76%)	2.7	27
Koren 2004 (ALLIANCE)	United States	1995–2002	2442	61.2	2007 (82.2%)	4.5	
Liem 2002 (FLORIDA)	Netherlands	1997–1999	540	60.5	448 (83%)	1	
**Mixed population**							
Amarenco 2006 (SPARCL)	Multi-centers	1998–2001	4731	62.7	2824 (59.7%)	4.9	27.4
Asselbergs 2004 (PREVEND IT)			864	51.3	562 (65%)	3.8	24.1
Blankenhorn 1993 (MARS)		1985–1989	247	58	225 (91%)	2.2	26
Chan 2010 (ASTRONOMER)	Canada	2002–2005	269	58	166 (61.7%)	3.5	28.1
Davis 2002 (ALLHAT-LTT)	North America	1994–2002	10355	66.4	5312 (51.3%)	4.8	29.9
Downs 1998 (AFCAPS/TexCAPS)	United States	1990–1993	6605	58	5608 (84.9%)	5.2	25
Emberson 2011 (MRC/BHF)	United Kingdom	1994–1997	20536	64	16244 (79.1%)	5	27.6
Fellstrom 2009 (AURORA)	25 countries	2003–2004	2773	64.2	1722 (62.1%)	3.8	25.4
Ford 2016 (WOSCOPS)	Scotland	1989–1991	6595	55	6595 (100%)	4.9	
Furberg 1994 (ACAPS)	United States	1989–1990	919	62	478 (52%)	2.8	25.9
Furberg 1995 (PLAC-I and -II)			559	58	159 (28.4%)	3	
Makuuchi 2005 (PCABG)	Japan	1991–1994	303	58.9	255 (84.2%)	4.5	23.7
Nakagawa 2004 (PCS)	Japan	1991–1995	120	55	109 (91.2%)	5.4	
Nakamura 2006 (MEGA)	Japan	1994–1999	7832	58.3	2428 (31%)	5.3	23.8
Ostadal 2010 (FACS)	Czech Republic	2003–2006	156	62.1	106 (68%)	1	
Pedersen 1994 (4S)	Scandinavia	1988–1989	4444	58.6	3617 (81.4%)	5.4	26
Ridker 2008 (JUPITER)	26 countries	2003–2006	17802	66	11002 (61.8%)	1.9	28.4
Riegger 1999	Germany and Czech Republic		365	59.8	225 (61.6%)	1	
Sawayama 2002 (FAST)	Japan	1996–2000	164	66.3	52 (31.7%)	2	23.2
Serruys 2002 (LIPS)	Europe, Canada, and Brazil	1996–1998	1677	60	1405 (83.8%)	3.9	26.6
Shepherd 2002 (PROSPER)	Ireland and Netherlands	1997–1999	5804	75.3	3825 (65.9%)	3.2	26.8
Takano 2013 (PEARL)	Japan	2006–2008	574	62.6	468 (81.5%)	3	
Tavazzi 2008 (GISSI-HF)	Italy	2002–2005	4574	68	3540 (77.4%)	3.9	27.1
Tonkin 1998 (LIPID)	Australia and New Zealand	1990–1992	9014	62	7482 (83%)	6.1	
Yokoi 2005 (ATHEROMA)	Japan	1994–1997	288	59.3	239 (83%)	3	
Yusuf 2016 (HOPE-3)	21 countries	2007–2010	12705	63.8	6835 (53.8%)	5.6	27.1
**Omega-3 supplementation**							
**Primary prevention**							
Andrieu 2017 (MAPT)	France and Monaco	2008–2011	1652	75.3	595 (36%)	3	26.1
Bhatt 2019 (REDUCE_IT)	11 countries	2011–2016	8159	64	5809 (71.2%)	4.9	
Bowman 2018 (ASCEND)	United Kingdom	2005–2011	15480	63.3	9690 (62.6%)	7.4	29.8
Roncaglioni 2013 (Risk and Prevention)	Italy	2004–2007	12505	64	7691 (61.5%)	5	30.8
Bosch 2012 (ORIGIN)	40 countries	2003–2005	12536	63.5	8148 (65%)	6.2	
**Secondary prevention**							
Kromhout 2010 (AlphaOmega)	Netherlands	2002–2006	4837	69	3783 (78.2%)	3.4	27.8
Tavazzi 2008 (GISSI-HF)	Italy	2002–2005	6975	68	5454 (78.2%)	3.9	27
**Mixed population**							
Bonds 2014 (AREDS2)	United States	2006–2008	4203	74	1816 (43.2%)	4.8	
Brouwer 2006 (SOFA)	European countries	2001–2004	546	61.5	459 (84.1%)	1	26.9
Dangour 2010 (OPAL)	England and Wales	2005–2006	867	75	477 (55%)	2	27.1
Einvik 2010 (DO IT)	Norwegian		563	70	563 (100%)	3	
Galan 2010 (SU.FOL.OM3)	France	2003–2007	2501	61.4	1986 (79.4%)	4.7	27.2
Macchia 2013 (FORWARD)		2008–2011	586	66.1	321 (54.8%)	1	
Manson 2019 (VITAL)	United States	2010–2018	25871	67.1	12780 (49.4%)	5.3	
Nilsen 2001	Norway	1995–1996	300	64	238 (79.3%)	1.5	26
Raitt 2005	United States	1999–2003	200	62.5	172 (86%)	2	
Rauch 2009 (OMEGA)	German	2003–2007	3804	64	2830 (74.4%)	1	27.5
Valagussa 1999 (GISSI-P)	Italy	1993–1995	11334	59.4	9668 (85.3%)	3.5	26.5
Yokoyama 2007 (JELIS)	Japan	1996–1999	18645	61	5855 (31.4%)	4.6	24

**Table A2:** History of cardiovascular events and risk factors of cardiovascular disease at baseline.

Study	Cardiovascular Disease	Coronary Heart Disease	Myocardial Infarction	Heart Failure	Hypertension	Dyslipidemia	Diabetes	Smoking	Obesity
**Statins**									
**Primary prevention**									
Bestehorn 1997 (CIS)								214 (84.3%)	
Blankenhorn 1993 (MARS)			148 (60%)		114 (46%)			198 (80%)	
Colhoun 2004 (CARDS)							2838 (100%)	1853 (65.3%)	1053 (37.1%)
Knopp 2006 (ASPEN)			395 (16.4%)		1328 (55.1%)	711 (29.5%)	2410 (100%)	299 (12.4%)	
Sever 2004 (ASCOT-LLA)					10305 (100%)		2535 (24.6%)	3370 (32.7%)	
Teo 2000 (SCAT)			322 (70%)		166 (36%)		51 (11%)	377 (82%)	
Wanner 2005			221 (17.6%)	444 (35.4%)			1255 (100%)	507 (40.4%)	
**Secondary prevention**									
Kjekshus 2007 (CORONA)			3007 (60%)	5011 (100%)	3157 (63%)		1478 (29.5%)	431 (8.6%)	
Koren 2004 (ALLIANCE)		2442 (100%)	1411 (57.8%)	161 (6.6%)			540 (22.1%)	476 (19.5%)	
Liem 2002 (FLORIDA)			540 (100%)						
**Mixed population**									
Amarenco 2006 (SPARCL)								2791 (59%)	
Arthros 2002 (GREACE)			1299 (81.2%)		686 (42.9%)		314 (19.6%)		
Asselbergs 2004 (PREVEND IT)	29 (3.4%)		3 (0.4%)	0 (0%)			22 (2.6%)	629 (72.8%)	
Chan 2010 (ASTRONOMER)								130 (48.3%)	
Davis 2002 (ALLHAT-LTT)		1470 (14.2%)					3635 (35.1%)	2402 (23.2%)	4401 (42.5%)
Downs 1998 (AFCAPS/TexCAPS)					1446 (21.9%)		152 (2.3%)	819 (12.4%)	
Emberson 2011 (MRC/BHF)		13677 (66.6%)	8933 (43.5%)				6243 (30.4%)	15361 (74.8%)	
Fellstrom 2009 (AURORA)	1104 (39.8%)		283 (10.2%)				732 (26.4%)	430 (15.5%)	
Ford 2016 (WOSCOPS)			0 (0%)					2902 (44%)	
Furberg 1994 (ACAPS)					265 (28.8%)			519 (56.5%)	
Furberg 1995 (PLAC-I and -II)			271 (48.5%)		226 (40.5%)		4 (0.7%)	87 (15.5%)	
Makuuchi 2005 (PCABG)			188 (62%)		156 (51.5%)		101 (33.3%)	127 (41.9%)	
Nakagawa 2004 (PCS)					71 (59.2%)	0 (0%)	21 (17.5%)	81 (67.5%)	
Nakamura 2006 (MEGA)		0 (0%)			3274 (41.8%)		1629 (20.8%)	1613 (20.6%)	
Ostadal 2010 (FACS)			12 (7.7%)		80 (51.3%)	18 (11.5%)	30 (19.2%)	46 (29.2%)	
Pedersen 1994 (4S)					1155 (26%)		200 (4.5%)	3324 (74.8%)	
Ridker 2008 (JUPITER)	0 (0%)	0 (0%)	0 (0%)	0 (0%)				2813 (15.8%)	
Riegger 1999			130 (35.6%)		107 (29.3%)	365 (100%)	20 (5.5%)	35 (9.6%)	
Sawayama 2002 (FAST)		68 (41.5%)			65 (39.6%)		41 (25%)	95 (57.8%)	
Serruys 2002 (LIPS)			745 (44.4%)		647 (38.6%)		201 (12%)	1199 (71.5%)	
Shepherd 2002 (PROSPER)	2565 (44.2%)		778 (13.4%)		3593 (61.9%)		621 (10.7%)	1555 (26.8%)	
Takano 2013 (PEARL)			144 (25.1%)		260 (45.3%)		157 (27.4%)		
Tavazzi 2008 (GISSI-HF)					2484 (54.3%)			645 (14.1%)	
Tonkin 1998 (LIPID)	1604 (17.8%)				3759 (41.7%)		784 (8.7%)	6607 (73.3%)	1614 (17.9%)
Yokoi 2005 (ATHEROMA)			131 (45.5%)		121 (42%)		54 (18.8%)		
Yusuf 2016 (HOPE-3)	0 (0%)	0 (0%)	0 (0%)	0 (0%)	4815 (37.9%)	4587 (36.1%)	737 (5.8%)	3519 (27.7%)	
**Omega-3 supplementation**									
**Primary prevention**									
Andrieu 2017 (MAPT)									
Bhatt 2019 (REDUCE_IT)						8159 (100%)	4781 (58.6%)		4683 (57.4%)
Bosch 2012 (ORIGIN)	7371 (58.8%)				9966 (79.5%)			1554 (12.4%)	
Bowman 2018 (ASCEND)	0 (0%)	0 (0%)	0 (0%)	0 (0%)	7647 (49.4%)		15480 (100%)	8328 (53.8%)	7198 (46.5%)
Roncaglioni 2013 (Risk and Prevention)				400 (3.2%)	10579 (84.6%)	8904 (71.2%)	7490 (59.9%)	2714 (21.7%)	6077 (48.6%)
**Secondary prevention**									
Kromhout 2010 (AlphaOmega)			4837 (100%)		4339 (89.7%)	4160 (86%)	1016 (21%)	817 (16.9%)	1171 (24.2%)
Tavazzi 2008 (GISSI-HF)			990 (14.2%)	6975 (100%)	3808 (54.6%)	1576 (22.6%)	1974 (28.3%)	990 (14.2%)	
**Mixed population**									
Bonds 2014 (AREDS2)	807 (19.2%)	147 (3.5%)	294 (7%)	147 (3.5%)	248 (5.9%)	1849 (44%)	546 (13%)	2379 (56.6%)	
Brouwer 2006 (SOFA)		384 (70.3%)	342 (62.6%)		277 (50.7%)		87 (15.9%)	366 (67%)	
Dangour 2010 (OPAL)			34 (3.9%)		485 (55.9%)				201 (23.2%)
Einvik 2010 (DO IT)	155 (27.5%)				158 (28%)	110 (19.5%)	82 (14.5%)	191 (34%)	
Galan 2010 (SU.FOL.OM3)			1150 (46%)					1821 (72.8%)	
Macchia 2013 (FORWARD)		69 (11.7%)		83 (14.1%)	536 (91.4%)	274 (46.7%)	76 (12.9%)	247 (42.2%)	
Manson 2019 (VITAL)				0 (0%)	12884 (49.8%)	9702 (37.5%)	3544 (13.7%)	1863 (7.2%)	
Nilsen 2001			70 (23.3%)	26 (8.7%)	73 (24.3%)		31 (10.3%)	227 (75.7%)	
Raitt 2005		146 (73%)	111 (55.5%)		101 (50.5%)	47 (23.5%)			
Rauch 2009 (OMEGA)			548 (14.4%)		2530 (66.5%)	1883 (49.5%)	1027 (27%)	1396 (36.7%)	
Valagussa 1999 (GISSI-P)			1360 (12%)		4035 (35.6%)		1677 (14.8%)	8750 (77.2%)	1643 (14.5%)
Yokoyama 2007 (JELIS)			1044 (5.6%)		6619 (35.5%)		3039 (16.3%)	3524 (18.9%)	

**Table A3:** Treatment arms and outcome events of studies included in the final analysis.

	Intervention Arm				Comparison Arm			
Study	Daily Dose	Sample Size	All-Cause Mortality	CVD Death	Daily Dose	Sample Size	All-Cause Mortality	CVD Death
**Statins**								
**Primary prevention**								
Bestehorn 1997 (CIS)	Simvastatin 40 mg	129		1 (0.8%)	Placebo	125		2 (1.6%)
Blankenhorn 1993 (MARS)	Lovastatin 80 mg	123	1 (0.8%)		Placebo	124	2 (1.6%)	
Colhoun 2004 (CARDS)	Atorvastatin 10 mg	1428	61 (4.3%)	134 (9.4%)	Placebo	1410	82 (5.8%)	189 (13.4%)
Knopp 2006 (ASPEN)	Atorvastatin 10 mg	1211	70 (5.8%)		Placebo	1199	68 (5.7%)	
Sever 2004 (ASCOT-LLA)	Atorvastatin 10 mg	5168	185 (3.6%)	74 (1.4%)	Placebo	5137	212 (4.1%)	82 (1.6%)
Teo 2000 (SCAT)	Simvastatin 10 mg	230	13 (5.7%)	7 (3%)	Placebo	230	6 (2.6%)	4 (1.7%)
Wanner 2005	Atorvastatin 20 mg	619	297 (48%)	121 (19.5%)	Placebo	636	320 (50.3%)	149 (23.4%)
**Secondary prevention**								
Kjekshus 2007 (CORONA)	Rosuvastatin 10 mg	2514	728 (29%)	581 (23.1%)	Placebo	2497	759 (30.4%)	593 (23.7%)
Koren 2004 (ALLIANCE)	Atorvastatin up to 80 mg	1217	121 (9.9%)	43 (3.5%)	Usual care	1225	127 (10.4%)	61 (5%)
Liem 2002 (FLORIDA)	Fluvastatin 80 mg	265	7 (2.6%)	6 (2.3%)	Placebo	275	11 (4%)	11 (4%)
**Mixed population**								
Amarenco 2006 (SPARCL)	Atorvastatin 80 mg	2365	216 (9.1%)	78 (3.3%)	Placebo	2366	211 (8.9%)	98 (4.1%)
Arthros 2002 (GREACE)	Atorvastatin 10-80 mg	800	23 (2.9%)		Usual care	800	40 (5%)	
Asselbergs 2004 (PREVEND IT)	Pravastatin 40 mg	433		4 (0.9%)	Placebo	431		4 (0.9%)
Chan 2010 (ASTRONOMER)	Rosuvastatin 40 mg	134		2 (1.5%)	Placebo	135		5 (3.7%)
Davis 2002 (ALLHAT-LTT)	Pravastatin 40 mg	5170	631 (12.2%)	295 (5.7%)	Usual care	5185	641 (12.4%)	300 (5.8%)
Downs 1998 (AFCAPS/TexCAPS)	Lovastatin 20 mg	3304		17 (0.5%)	Placebo	3301		25 (0.8%)
Emberson 2011 (MRC/BHF)	Simvastatin 40 mg	10269	1328 (12.9%)		Placebo	10267	1507 (14.7%)	
Fellstrom 2009 (AURORA)	Rosuvastatin 10 mg	1389	636 (45.8%)	324 (23.3%)	Placebo	1384	660 (47.7%)	324 (23.4%)
Ford 2016 (WOSCOPS)	Pravastatin 40 mg	3302	619 (18.7%)	252 (7.6%)	Placebo	3293	674 (20.5%)	297 (9%)
Furberg 1994 (ACAPS)	Lovastatin 20-40 mg (± warfarin 1 mg)	460	1 (0.2%)		Placebo (± warfarin 1 mg)	459	8 (1.7%)	
Furberg 1995 (PLAC-I and -II)	Pravastatin 20 or 40 mg	281	7 (2.5%)	5 (1.8%)	Placebo	278	11 (4%)	5 (1.8%)
Makuuchi 2005 (PCABG)	Pravastatin 10-20 mg	152	6 (3.9%)	4 (2.6%)	No lipid lowering agent	151	11 (7.3%)	4 (2.6%)
Nakagawa 2004 (PCS)	Pravastatin 10 mg	54	17 (31.5%)	2 (3.7%)	Dietary control	66	23 (34.8%)	1 (1.5%)
Nakamura 2006 (MEGA)	Pravastatin 10-20 mg	3866	55 (1.4%)	11 (0.3%)	Dietary control	3966	79 (2%)	18 (0.5%)
Ostadal 2010 (FACS)	Fluvastatin 80 mg	78		1 (1.3%)	Placebo	78		4 (5.1%)
Pedersen 1994 (4S)	Simvastatin 20 mg	2221	182 (8.2%)	136 (6.1%)	Placebo	2223	256 (11.5%)	207 (9.3%)
Ridker 2008 (JUPITER)	Rosuvastatin 20 mg	8901	198 (2.2%)		Placebo	8901	247 (2.8%)	
Riegger 1999	Fluvastatin 40 or 80 mg	187		2 (1.1%)	Placebo	178		4 (2.2%)
Sawayama 2002 (FAST)	Pravastatin 10 mg	83	5 (6%)		Dietary control	81	9 (11.1%)	
Serruys 2002 (LIPS)	Fluvastatin 80 mg	844	36 (4.3%)	13 (1.5%)	Placebo	833	49 (5.9%)	24 (2.9%)
Shepherd 2002 (PROSPER)	Pravastatin 40 mg	2891	298 (10.3%)		Placebo	2913	306 (10.5%)	
Takano 2013 (PEARL)	Pitavastatin 2 mg	288	27 (9.4%)	24 (8.3%)	No statin	286	37 (12.9%)	22 (7.7%)
Tavazzi 2008 (GISSI-HF)	Rosuvastatin 10 mg	2285	657 (28.8%)	478 (20.9%)	Placebo	2289	644 (28.1%)	488 (21.3%)
Tonkin 1998 (LIPID)	Pravastatin 40 mg	4512	498 (11%)	331 (7.3%)	Placebo	4502	633 (14.1%)	433 (9.6%)
Yokoi 2005 (ATHEROMA)	Pravastatin 10-20 mg + diet	142	1 (0.7%)		Dietary control	146	2 (1.4%)	
Yusuf 2016 (HOPE-3)	Rosuvastatin 10 mg	6361	334 (5.3%)	154 (2.4%)	Placebo	6344	357 (5.6%)	171 (2.7%)
**Omega-3 supplementation**								
**Primary prevention**								
Andrieu 2017 (MAPT)	EPA + DHA up to 300/1300 mg	820	20 (2.4%)		Placebo	832	20 (2.4%)	
Bhatt 2019 (REDUCE_IT)	Icosapent ethyl 4 g	4069	274 (6.7%)	174 (4.3%)	Placebo	4090	310 (7.6%)	213 (5.2%)
Bosch 2012 (ORIGIN)	EPA + DHA 465/375 mg	6281	951 (15.1%)	574 (9.1%)	Olive oil	6255	964 (15.4%)	581 (9.3%)
Bowman 2018 (ASCEND)	EPA + DHA 460/380 mg	7740	752 (9.7%)	186 (2.4%)	Olive oil	7740	788 (10.2%)	228 (2.9%)
Roncaglioni 2013 (Risk and Prevention)	EPA + DHA 1 g (ratio 0.9:1 to 1.5:1)	6239	348 (5.6%)	142 (2.3%)	Olive oil	6266	337 (5.4%)	137 (2.2%)
**Secondary prevention**								
Kromhout 2010 (AlphaOmega)	EPA+DHA 400 mg (± ALA 2 g)	2404	186 (7.7%)	80 (3.3%)	Control (± ALA 2 g)	2433	184 (7.6%)	82 (3.4%)
Tavazzi 2008 (GISSI-HF)	EPA + DHA 850 to 882 mg	3494	2157 (61.7%)	712 (20.4%)	Placebo	3481	2202 (63.3%)	765 (22%)
**Mixed population**								
Bonds 2014 (AREDS2)	EPA + DHA 650/350 mg	2147	200 (9.3%)	14 (0.7%)	Placebo	2056	168 (8.2%)	13 (0.6%)
Brouwer 2006 (SOFA)	EPA + DHA 464/335 mg	273	8 (2.9%)	6 (2.2%)	Sunflower oil	273	14 (5.1%)	13 (4.8%)
Einvik 2010 (DO IT)	EPA + DHA 1176/840 mg	282	14 (5%)	7 (2.5%)	Placebo	281	24 (8.5%)	11 (3.9%)
Galan 2010 (SU.FOL.OM3)	EPA + DHA 400/200 mg	1253	58 (4.6%)		Placebo	1248	59 (4.7%)	
Dangour 2010 (OPAL)	EPA + DHA 400/1000 mg	434	9 (2.1%)		Placebo	433	8 (1.8%)	
Macchia 2013 (FORWARD)	EPA + DHA 850 to 882 mg	289	4 (1.4%)		Olive oil	297	5 (1.7%)	
Manson 2019 (VITAL)	EPA + DHA 460/380 mg	12933	371 (2.9%)	142 (1.1%)	Placebo	12938	381 (2.9%)	148 (1.1%)
Nilsen 2001	EPA + DHA 850 to 882 mg (ratio 1:2)	150	11 (7.3%)	8 (5.3%)	Corn oil	150	11 (7.3%)	8 (5.3%)
Raitt 2005	EPA + DHA 756/540 mg	100	4 (4%)	2 (2%)	Placebo	100	10 (10%)	5 (5%)
Rauch 2009 (OMEGA)	EPA + DHA 460/380 mg	1919	88 (4.6%)	67 (3.5%)	Olive oil	1885	70 (3.7%)	51 (2.7%)
Valagussa 1999 (GISSI-P)	EPA + DHA 850 to 882 mg (± vitamin E 300 mg)	5666	472 (8.3%)	291 (5.1%)	Control (± vitamin E 300 mg)	5668	545 (9.6%)	348 (6.1%)
Yokoyama 2007 (JELIS)	EPA 1800 mg + statin	9326	286 (3.1%)		Statin only	9319	265 (2.8%)	

CVD, cardiovascular disease; EPA, eicosapentaenoic acid; DHA, docosahexaenoic acid; ALA, alpha-linolenic acid.

**Table A4:** Dose–response effects of statins and omega-3 supplementation on all-cause mortality and cardiovascular death.

All-Cause Mortality			Cardiovascular Death		
Intervention	RR (95% CI)	*p*-Value	Intervention	RR (95% CI)	*p*-Value
Atorvastatin	0.97 (0.93–1.01)	0.15	Atorvastatin	**0.90 (0.83–0.98)**	0.02
Pravastatin	0.97 (0.95–1.00)	0.05	Pravastatin	**0.96 (0.94–0.98)**	0.001
Simvastatin	0.93 (0.80–1.07)	0.32	Simvastatin	**0.82 (0.74–0.91)**	< 0.001
Rosuvastatin	**0.91 (0.80–0.99)**	0.02	Rosuvastatin	0.97 (0.91–1.03)	0.36
Lovastatin	0.93 (0.40-1.30)	0.28	Fluvastatin	**0.92 (0.86–0.98)**	0.01
Omega-3 supplementation	0.98 (0.95–1.01)	0.11	Omega-3 supplementation	**0.94 (0.89–0.99)**	0.01

RR, relative risk; CI, confidence interval. Data are presented as the effect of per increment in 10 mg of statins and 1000 mg of omega-3 supplementation. Bold font indicates statistical significance.
